# Epigenetic effects of pharmacologic ascorbate

**DOI:** 10.18632/oncotarget.27911

**Published:** 2021-04-27

**Authors:** Garett J. Steers, Rory S. Carroll, Brianne R. O’Leary, Joseph J. Cullen

**Keywords:** pancreatic cancer, pharmacological ascorbate, dual oxidases, catalase, hydrogen peroxide

Pancreatic cancer continues to carry a grim prognosis with a 5-year overall survival of 10% but a dismal 3% with metastatic disease, numbers that have changed little over the last decade despite advances in chemotherapy [1]. This is due to a number of factors including advanced stage at diagnosis and resistance to radio-chemotherapy. Recently, pharmacologic ascorbate (ascorbic acid, ascorbate, P-AscH^-^) given at high intravenous doses has been investigated as an adjuvant to chemotherapy and radiation therapy in the treatment of pancreatic cancer. P-AscH^-^ has been shown to act as an oxidizing agent at high doses, generating hydrogen peroxide (H_2_O_2_) to cancer cells resulting in selective cytotoxicity while protecting normal tissue [2–8]. Recent studies have shown P-AscH^-^ also suppresses cancer cell growth through epigenetic mechanisms, namely DNA demethylation [9].

DNA hypermethylation and subsequent decreased gene expression has been observed across multiple malignancies including acute myeloid leukemia, hepatocellular carcinoma, lung cancer, colorectal cancer, and ovarian cancer [10–14]. Decreased expression of tumor suppressor genes is believed to mediate this process. In addition to generating reactive oxygen species, P-AscH^-^ has an epigenetic role as a cofactor for the ten-elevation translocation (TET) methylcytosine dioxygenase family of enzymes, responsible for DNA demethylation primarily at gene promotor regions resulting in increased downstream gene expression [15, 16]. One such family of genes that has been identified and characterized in several malignancies is the dual oxidase (DUOX1 and DUOX2) enzymes of the NADPH oxidase family.

The NADPH oxidase (NOX) family of enzymes is defined by their ability to produce reactive oxygen species (ROS) [17]. Out of the seven members of this group, DUOX1 and DUOX2 are unique in that they specifically only produce H_2_O_2_, and in fact are activated by H_2_O_2_ [18]. The DUOX enzymes have been reported in epithelial tissues, including the pancreas, and may play a role in cancer progression and response to therapy in pancreatic cancer. For example, decreased DUOX expression has been shown to increase epithelial to mesenchymal transition (EMT), while DUOX restoration decreases cancer cells’ ability to grow colonies *in vitro* [11]. Although the downregulation of DUOX1 and DUOX2 has been implicated in many cancers where their downregulation correlates with decreased overall survival, their role in pancreatic cancer was largely unknown [10–12, 19]. Recently our group demonstrated significant downregulation of both DUOX1 and DUOX2 across multiple pancreatic cell lines [20]. Baseline mRNA levels of the DUOX enzymes were increased in pancreatic cancer cells after treatment with P-AscH^-^ in a dose- and time-dependent manner, but unchanged in normal pancreatic ductal epithelial cells. In addition, there were sustained increases in ROS 48 hours after just one hour of treatment with P-AscH^-^. These increases were reversed with the addition of catalase, suggesting the sustained effects were dependent on the initial increase of H_2_O_2_ induced by P-AscH^-^. To determine the source of this increased oxidation, a mitochondrial stress test demonstrated increases in non-mitochondrial respiration, supporting the hypothesis that the NOX family enzymes may contribute to this late, sustained oxidative stress. Inhibiting the DUOX enzymes partially reversed P-AscH^-^ -induced cytotoxicity, suggesting P-AscH^-^-induced increases in DUOX enzymes contributes to its cytotoxic effects on pancreatic cancer cells. P-AscH^-^ may mediate these effects *via* two pathways including ascorbate oxidation leading to generation of H_2_O_2_ and epigenetic enhancement of TET, leading to DNA demethylation and increased gene expression including DUOX1 and DUOX2 resulting in sustained H_2_O_2_ production.

An alternative pathway to inducing DNA demethylation is *via* inhibition of the DNA methyltransferase (DNMT) family of enzymes, responsible for the methylation of CpG base pairs in DNA promoter regions. DNMT1 and DNMT3 are overexpressed in multiple cancers including pancreatic cancer [21]. DNMT inhibitors such as azacytidine and decitabine, which act as cytidine analogs, are commonly utilized in the treatment of myelodysplastic syndrome and acute myeloid leukemia [22–25]. These therapies have been studied in multiple solid organ malignancies, and in fact have been shown to increase immunotherapy effectiveness in pancreatic cancer models [26]. However, they have not been adopted into standard of care for solid malignancies. DNMT inhibitors could possibly have similar effects as P-AscH^-^ on the expression of DUOX in pancreatic cancer leading to increased H_2_O_2_ production and cytotoxicity. Additionally, P-AscH^-^ in combination with a DNMT inhibitor may offer a therapeutic advantage in the treatment of pancreatic cancer through both direct cytotoxic mechanisms and epigenetic alterations.
